# Growth Promotion or Osmotic Stress Response: How SNF1-Related Protein Kinase 2 (SnRK2) Kinases Are Activated and Manage Intracellular Signaling in Plants

**DOI:** 10.3390/plants10071443

**Published:** 2021-07-15

**Authors:** Yoshiaki Kamiyama, Sotaro Katagiri, Taishi Umezawa

**Affiliations:** 1 Graduate School of Bio-Applications and Systems Engineering, Tokyo University of Agriculture and Technology, Koganei, Tokyo 184-8588, Japan; s197730t@st.go.tuat.ac.jp (Y.K.); s210006x@st.go.tuat.ac.jp (S.K.); 2 Faculty of Agriculture, Tokyo University of Agriculture and Technology, Fuchu, Tokyo 183-8538, Japan

**Keywords:** abscisic acid, osmotic stress, protein kinase, growth promotion, SnRK2, Raf

## Abstract

Reversible phosphorylation is a major mechanism for regulating protein function and controls a wide range of cellular functions including responses to external stimuli. The plant-specific SNF1-related protein kinase 2s (SnRK2s) function as central regulators of plant growth and development, as well as tolerance to multiple abiotic stresses. Although the activity of SnRK2s is tightly regulated in a phytohormone abscisic acid (ABA)-dependent manner, recent investigations have revealed that SnRK2s can be activated by group B Raf-like protein kinases independently of ABA. Furthermore, evidence is accumulating that SnRK2s modulate plant growth through regulation of target of rapamycin (TOR) signaling. Here, we summarize recent advances in knowledge of how SnRK2s mediate plant growth and osmotic stress signaling and discuss future challenges in this research field.

## 1. Introduction

Throughout their lifecycles, plants are sometimes exposed to water-deficit conditions such as drought, salinity and freezing. Such conditions are perceived by plant tissues as an osmotic stress that reduces plant growth, leading to losses in crop yield worldwide. To cope with such conditions and maintain cellular homeostasis, plant cells have developed physiological and molecular mechanisms to recognize and respond to external stresses in a precise and timely manner. Such external stimuli are generally perceived at the plasma membrane of cells and then transmitted to the entire cell through various signaling pathways. Protein phosphorylation is a post-translational modification that plays an important role in the cellular response to osmotic stress. Phosphorylation is catalyzed by protein kinases, which transfer a γ- phosphoryl group from ATP to specific serine, threonine, tyrosine or sometimes histidine residues within their substrate proteins [1]. Phosphorylation can affect the stability, catalytic activity and subcellular localization of proteins, as well as interactions with other regulatory components [2,3]. This modification is considered reversible because phosphorylated proteins can also be readily dephosphorylated by protein phosphatases [4]. Of the ~28,000 protein-encoding genes in the model plant *Arabidopsis thaliana* [5], over 1000 genes encode protein kinases [6] and only a small subset of these genes have been functionally characterized to date.

The collective effort by many researchers over the past two decades has resulted in a deeper understanding of the essential roles of a phytohormone abscisic acid (ABA) and SnRK2-type protein kinases in the plant responses to diverse environmental stimuli, especially to osmotic stress [7,8]. ABA plays crucial roles in plant responses to osmotic stress and desiccation, such as stomatal closure, gene expression, seed maturation and dormancy [9,10]. When exposed to water-deficit conditions, plants accumulate higher levels of ABA, which in turn induces protective stress responses via ABA-dependent or -independent pathways, both of which trigger activation of multiple SnRK2s [11,12,13]. Following their activation, SnRK2s transduce the stress signal through phosphorylation of various downstream substrates, including transcription factors and ion channels. In turn, these downstream substrates facilitate protective stress responses [14,15]. Although the mechanisms by which ABA induces SnRK2 activation are well characterized [16,17,18,19], how osmotic stress induces direct activation of SnRK2s was, until recently, less clear. A breakthrough came in 2015, when a pioneering study identified a group B3 Raf-like protein kinase as a direct upstream activator of SnRK2s in the moss [20]. Following this initial study, several additional studies revealed the essential roles of group B Raf-like protein kinases in ABA-independent SnRK2 activation in response to osmotic stress responses in Arabidopsis [21,22,23,24]. In addition to these new connections between SnRK2s and Raf-like kinases, recent data shows that SnRK2-mediated signaling also regulates target of rapamycin (TOR), a master regulator that orchestrates cell proliferation and growth by integrating nutrient, energy, hormone and diverse stress stimuli [25,26,27]. Together, these studies indicate that SnRKs play a pivotal role in the balance between plant growth and stress responses.

Here, we summarize recent knowledge of how SnRK2-mediated signaling contributes to plant growth and osmotic stress responses, with a focus on how SnRK2 activity is regulated by upstream signaling components in ABA-dependent or -independent manner and how SnRK2s transmit signals to downstream signaling factors to induce protective stress responses under osmotic stress conditions. We also highlight how SnRK2s function to maintain a balance between growth promotion and stress responses.

## 2. Role of SnRK2 Protein Kinases in Plant Abiotic Stress Tolerance

**Figure 1:**
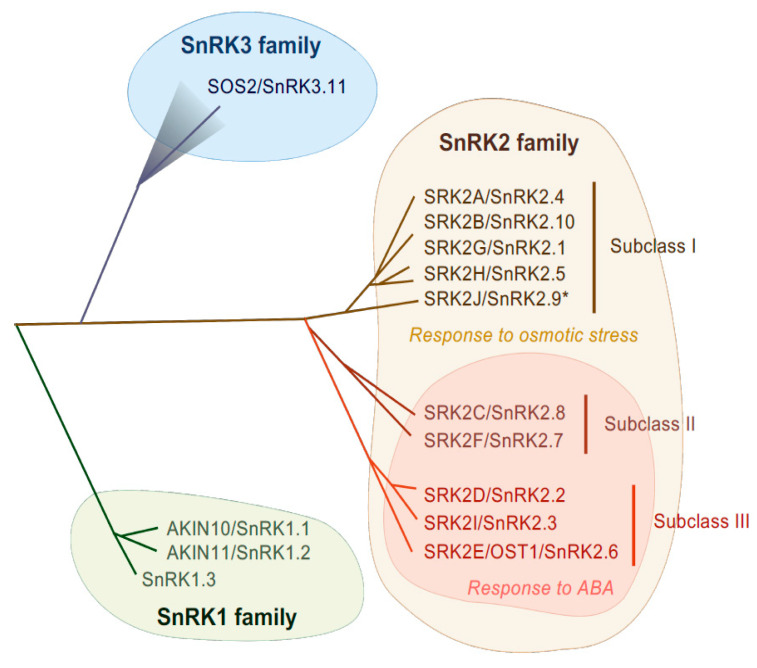
Phylogenetic tree of SnRK family in Arabidopsis. In Arabidopsis, SnRK1, SnRK2 and SnRK3 contain 3, 10 and 25 genes, respectively. SnRK2 family is classified into three subclasses, subclass I, II and III. SnRK2 kinases are activated in response to osmotic stress, except for SRK2J (indicated by *). Subclass II and Subclass III SnRK2s are activated in response to ABA.

SnRK2-type protein kinases belong to the SnRK family and are classified into three subgroups (SnRK1, SnRK2 and SnRK3) according to their sequence similarity and C-terminal domain structure [28] (Figure 1). The heterotrimeric SnRK1s are the most closely related to yeast SUCROSE NON-FERMENTING 1 (SNF1) kinase and mammalian AMP-ACTIVATED PROTEIN KINASEs (AMPKs) [28] and are involved in cellular responses to starvation or nutrient signals [29]. Differing from SnRK1, the SnRK2 and SnRK3 groups are unique to plants. SnRK3 is also named as CALCINEURIN B-LIKE (CBL)-INTERACTING PROTEIN KINASE (CIPK) according to their ability of interacting with Ca^2+^ sensor CBL proteins, or PROTEIN KINASE S (PKS). The most characterized SnRK3 kinase is SALT OVERLY SENSITIVE 2 (SOS2), also known as SnRK3.11/CIPK24, that is a serine/threonine kinase and that forms a complex with CBL4/SOS3. The SOS2-SOS3 complex acts in the SOS pathway that is required for salt tolerance [30]. SnRK2 families are evolutionarily conserved in major plant lineages, including algae (e.g., *Klebsormidium nitens* and *Chlamydomonas reinhardtii*), liverwort (*Marchantia polymorpha*), moss (*Physcomitrella patens*), lycophytes (*Selaginella moellendorffii*) and land plants (*Arabidopsis thaliana* and *Oryza sativa*) [31,32,33]. The first reported SnRK2 was PROTEIN KINASE ABA 1 (PKABA1) from wheat and its transcript was induced by ABA [34]. Later, ABA-ACTIVATED PROTEIN KINASE (AAPK) was found from fava bean (*Vicia fava*) [35] and its Arabidopsis ortholog OPEN STOMATA 1 (OST1)/ SRK2E/ SnRK2.6 was identified [36,37].

SnRK2s are monomeric serine/threonine protein kinases with a molecular mass of about 40 kDa [38], consisting of an N-terminal protein kinase domain and a C-terminal domain that contains stretches of acidic amino acids (acidic patch). The C-terminal domains are further functionally categorized as domain I and domain II, also known as SnRK2-box or ABA-box, respectively [11,39,40]. The amino acid sequences encoding the N-terminal kinase domain are highly conserved among all SnRK2 members, while sequences encoding the C-terminal domain are more divergent [11,28,36]. These C-terminal domains are required for SnRK2 activation in a stress-specific manner, with the SnRK2-box necessary for osmostress-induced or ABA-independent activation and the ABA-box necessary for ABA-dependent activation [11,13,39]. On the basis of the amino acid sequence similarity and responsiveness to ABA or osmotic stress, the SnRK2 family can be classified into three subclasses: I, II and III [12,13] (Figure 1). Although SnRK2-box is relatively similar in all SnRK2 members, the ABA-box is apparently different among SnRK2 subclasses, especially within the acidic patch region. Indeed, the acidic patch is rich in glutamic acid (E) in subclass I SnRK2s, but rich in aspartic acid (D) in subclass II and III [11]. Although only D-rich SnRK2s (subclass II and III) are activated by ABA, all SnRK2 members except for Arabidopsis SRK2J/SnRK2.9 (subclass I) are activated in response to osmotic stress [11,12,13] (Figure 1). Thus, subclass II and III SnRK2s are classified as ABA-responsive SnRK2s, while subclass I SnRK2s are classified as ABA-unresponsive SnRK2s.

### 2.1. Role of Arabidopsis Subclass III SnRK2s in Regulating Abiotic Stress Responses

The Arabidopsis genome contains ten members of the SnRK2 family, designated SRK2A to SRK2J [36] or SnRK2.1 to SnRK2.10 [28] (Figure 1). Among these subclasses, subclass III members (SRK2D/SnRK2.2, SRK2E/SnRK2.6/OST1 and SRK2I/SnRK2.3) are strongly activated by ABA and osmotic stress treatment and play essential roles in the induction of multiple osmotic stress responses including activation of ABA signaling [12]. SRK2E is expressed in guard cells dominantly and plays a key role in stomatal movement [36,37], while SRK2D and SRK2I are mainly involved in ABA signaling during seed germination, seed dormancy and seedling growth [41]. The triple knockout mutant for subclass III SnRK2s (*srk2dei*) showed extreme insensitivity to ABA [18,42,43,44], indicating that those three kinases are functionally redundant.

**Figure 2:**
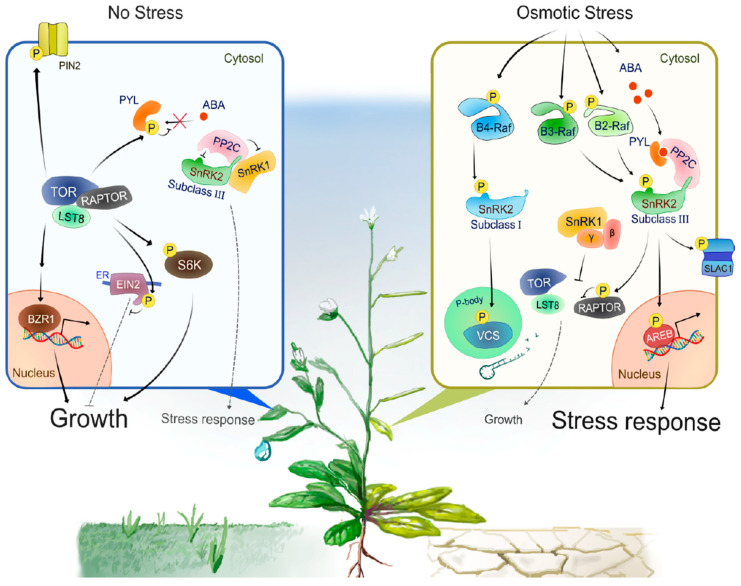
A model of the intracellular signal transduction under optimal growth conditions or during osmotic stress responses.Under optimal growth conditions (No Stress), TOR, RAPTOR and LST8 form a complex and promote cell division, expansion or elongation for plant growth through positive regulation of growth promotion-related factors such as S6K, BZR1 and PIN2. In addition, TOR directly phosphorylates PYLs or EIN2 to negatively regulates stress-associated phytohormone signaling such as ABA and ethylene, respectively. TOR-mediated phosphorylation of PYLs inhibits PYL-binding to ABA, therefore active PP2Cs dephosphorylate and inactivate subclass III SnRK2s to compromise ABA signaling and other protective stress responses. Such a PP2C-SnRK2 complex sequesters SnRK1, thereby allowing TOR activity and growth. Under water-deficit conditions (Osmotic Stress), multiple group B Raf-like protein kinases are rapidly activated. B4 Raf-like protein kinases directly phosphorylate and activate ABA-unresponsive (subclass I) SnRK2s, thereby enhancing mRNA decapping activator VARICOSE (VCS) activity to modulate mRNA population under osmotic stress responses. On the other hand, ABA-responsive (subclass III) SnRK2s activate in the both ABA-dependent and -independent manner. That is, osmotic stress promotes ABA biosynthesis and increased ABA is perceived by PYLs, leading to the formation of PYL-ABA-PP2C complexes, thereby releasing subclass III SnRK2s from inhibition by PP2Cs. In addition, osmostress-activated B2/B3 Raf-like protein kinases directly phosphorylate and activate subclass III SnRK2s. Activated subclass III SnRK2s phosphorylate downstream substrates such as bZIP-type transcription factor AREBs and ion channel SLAC1, to promote ABA responses or other protective stress responses including regulation of stress-responsive gene expression and stomatal closure. SnRK1 is released from the sequestration by PP2C-SnRK2 complex and inhibits TOR-mediated signaling to repress growth. The β and γ represent the regulatory subunits of heterotrimeric SnRK1 complex.

Progress has been made in identifying proteins phosphorylated by subclass III SnRK2s. For example, bZIP-type transcription factors AREB/ABFs (ABA-RESPONSIVE ELEMENT-BINDING PROTEINS/ABRE-BINDING FACTORS) are phosphorylated by SnRK2s to induce ABA- or stress-responsive gene expression [45,46] (Figure 2). In guard cells, SRK2E phosphorylates various membrane-localized proteins to induce stomatal closure and reduce transpiration. Activation of anion channels SLOW ANION CHANNEL-ASSOCIATED 1 (SLAC1) [47,48] and QUICK-ACTIVATING ANION CHANNEL 1 (QUAC1) [49] by SRK2E induces anion efflux through the plasma membrane, leading to activation of outward-rectifying K^+^ channel GATED OUTWARDLY-RECTIFYING K^+^ CHANNEL (GORK) [50] and K^+^ release. Another SRK2E target is the inward-rectifying K^+^ channel POTASSIUM CHANNEL IN ARABIDOPSIS THALIANA 1 (KAT1). SRK2E phosphorylates and inactivates KAT1 to prevent K^+^ influx [51]. Additionally, SRK2E phosphorylates a NADPH oxidase RESPIRATORY BURST OXIDASE HOMOLOG F (RbohF) to promote apoplast reactive oxygen species (ROS) production [52]. The accumulation of apoplastic ROS is crucial for the induction of stomatal closure. More recent comparative phosphoproteomic analyses identified additional putative substrates downstream of subclass III SnRK2s [14,15]. Among these substrates, SNRK2-SUBSTRATE 1 (SNS1) negatively regulates ABA signaling at cotyledon greening stage [14], whereas SERRATE (SE) and HYPONASTIC LEAVES 1 (HYL1), core components of miRNA processing complex, are phosphorylated by SnRK2s to modulate miRNA accumulation [15,53]. The global view provided by these phosphoproteomic approaches also revealed [-(R/K)-x-x-(*p*S/*p*T)-] and [-(*p*S/*p*T)-x-x-x-x-(D/E)-] as the preferred motifs that SnRK2s phosphorylate within their substrates in Arabidopsis [14].

There are several notable differences in the composition of SnRK2 in Arabidopsis compared to other plants. The subclass III-type SnRK2s are the most ancestral SnRK2 kinases that can be found in semiterrestrial algae [32,54]. The moss *Physcomitrella patens* genome encodes only four *SnRK2* genes (*PpSnRK2A*/*2B*/*2C*/*2D*), all of which are categorized into subclass III [55]. The *Ppsnrk2a*/*b*/*c*/*d* quadruple knockout mutant displayed drastic ABA insensitivity and reduced osmotic stress tolerance, indicating that the roles of subclass III SnRK2s in abiotic stress signaling are evolutionarily conserved between bryophytes and angiosperms [54].

### 2.2. Role of Arabidopsis Subclass I and II SnRK2s in Regulating Abiotic Stress Responses

In Arabidopsis, there are two subclass II SnRK2s (SRK2C/SnRK2.8 and SRK2F/SnRK2.7), both of which are strongly activated in response to osmotic stress but weakly to ABA [12] (Figure 1). The first reported SnRK2, PKABA1 from wheat, belongs to this subclass [13,34]. Overexpression of *SRK2C* confers drought tolerance in Arabidopsis transgenic plants [56]. However, by contrast to the severe phenotype observed in subclass III mutant (*srk2dei*), the Arabidopsis double mutant (*srk2cf*) did not showed remarkable phenotypic changes even under osmotic stress conditions [33], indicating only minor contributions to ABA and osmostress signaling. bZIP-type transcription factors, such as ABF3 and ENHANCED EM LEVEL (EEL), are suggested to be as putative SRK2C substrates in vitro [33]. On the other hand, SRK2C was shown to function in metabolic processes, suggesting its crucial roles in plant growth [57]. Given that subclass II-type SnRK2 has been acquired in lycophytes (e.g., *Selaginella tamariscina*), it could be an intermediate molecule during the transition from subclass III SnRK2 in algae to subclass I SnRK2 in seed plants [33].

Subclass I-type SnRK2s are found in most angiosperms, but not in bryophytes or algae. There are five members in the Arabidopsis genome (SRK2A/SnRK2.4, SRK2B/SnRK2.10, SRK2G/SnRK2.1, SRK2H/SnRK2.5 and SRK2J/SnRK2.9), all of which except for SRK2J are rapidly activated by osmotic stress perception prior to ABA accumulation [11,12] (Figure 1). Unlike subclass II and III SnRK2s, subclass I SnRK2s are not activated by ABA [11,12] (Figure 2). Although less is known about subclass I SnRK2s compared to subclass III SnRK2s, recent studies have shown that this clade of SnRK2 is also essential for plant growth and survival under water-deficit conditions. For example, under salt stress conditions, SRK2A and its homolog SRK2B are involved in the maintenance of root system architecture [58] and in the modulation of ROS homeostasis [59]. Additionally, under osmotic stress conditions, SRK2B phosphorylates two of the LATE EMBRYOGENESIS ABUNDANT (LEA) dehydrin proteins, EARLY RESPONSE TO DEHYDRATION 10 (ERD10) and ERD14 [60]. Phosphorylation of ERD14 by SRK2B are involved in the translocation of ERD14 from cytosol to nucleus [60]. Importantly, after osmotic stress perception, subclass I-type SnRK2s, such as SRK2A and SRK2G, translocate to cytosolic punctate structures, which is known as processing bodies (P-bodies) [58,61]. In P-bodies, subclass I SnRK2s interact with and phosphorylate the mRNA decapping activator VARICOSE (VCS) [61] (Figure 2). VCS is a component of the mRNA decapping complex, which mediates the removal of the mRNA 5′-m^7^G-cap, leading to exonuclease-mediated mRNA decay [62]. Both the quadruple knockout mutant of subclass I SnRK2s (*srk2abgh*) and artificial micro RNA (amiRNA)-mediated *VCS*-knockdown plants shows similar growth retardation under osmotic stress conditions, suggesting the common phenotype is due to misregulation of mRNA metabolism in response to osmotic stress [61]. Given the fact that ABA-responsive SnRK2s (subclass II and III) showed no interaction with VCS [61], subclass I SnRK2s could predominantly regulate VCS-mediated mRNA decay in early osmotic stress response. In addition, cross-species complementation attempts demonstrated that Arabidopsis subclass I SnRK2 could not complement the osmotic stress tolerance-related phenotype of the *P. patens* subclass III SnRK2s quadruple mutant (*Ppsnrk2a*/*b*/*c*/*d*), indicating that the functions of subclass I SnRK2s are not compatible with subclass III SnRK2s [54]. In conclusion, recent investigations proposed that subclass I SnRK2s could be involved in plant growth and osmotic stress tolerance in a different manner from subclass II/ III SnRK2s, but both subclass I SnRK2-mediated and subclass II/ III SnRK2-mediated osmostress signaling are vital for plant to adopt to unfavorable conditions (Figure 2).

### 2.3. Other Regulators of Abiotic Stress Responses

In addition to the SnRK2 family, other protein kinases such as RECEPTOR-LIKE KINASES (RLKs), MITOGEN-ACTIVATED PROTEIN KINASES (MAPKs), CALCIUM-DEPENDENT PROTEIN KINASES (CDPKs/CPKs) contribute to plant responses to various abiotic stresses. For example, a plasma membrane localized receptor-like kinase GUARD CELL HYDROGEN PEROXIDE-RESISTANT 1 (GHR1) phosphorylates SLAC1 and is involved in both ABA- and H_2_O_2_-mediated stomatal closure [63]. Another transmembrane receptor-like kinase FERONIA (FER) and its ligand RAPID ALKALINIZATION FACTOR 1 (RALF1) interact with and activate the GTPase RHO-RELATED PROTEIN FROM PLANTS 11 (ROP11), thereby activating PP2C ABI2 to suppress ABA signaling [64]. RALF1-FER complex-dependent phosphorylation of GLYCINE-RICH RNA BINDING PROTEIN 7 (GRP7) affects alternative splicing to modulate stress responses and growth [65]. MPK9 and MPK12 are highly and preferentially expressed in guard cells and are involved in both ABA- and H_2_O_2_-mediated stomatal closure [66]. ABA-inducible MAPKKK17/18 activates MAPKK3–MPK1/2/7/14 cascade to regulate ABA-mediated inhibition of seed germination and cotyledon greening [67]. MAPKKK20, also known as ABA-INSENSITIVE PROTEIN KINASE 1 (AIK1), activates MAPKK5–MPK6 cascade to promote ABA-mediated primary root growth inhibition and stomatal closure and the PP2C ABI1 restricts AIK1 activity by dephosphorylation [68]. CPKs also mediate abiotic stress signaling through phosphorylation of substrate proteins, some of which are partially overlapped with SnRK2s. One of the examples is CPK6, which directly phosphorylates the N-terminal region of SLAC1 at Ser-59 and activates SLAC1 to promote stomatal closure [69]. Notably, transgenic plants carrying a *SLAC1* transgene with mutations that produce a single substitution of Ser-59 to Ala (S59A), or the OST1-target site Ser-120 to Ala (S120A), rescued the *slac1* knockout mutant phenotype. However, a transgenic line carrying a *SLAC1* transgene with mutations that produce both Ser to Ala substitutions did not, suggesting that both CPK6- and OST1-mediated phosphorylation is essential for SLAC1 activation [69]. In addition to those, several protein kinases are modulating plant responses to abiotic stresses, which have been extensively discussed in the excellent recent reviews [70,71].

## 3. Molecular Mechanisms of SnRK2 Activation

### 3.1. ABA Signaling Pathway

As described above, a subset of subclass III SnRK2s, with C-terminal regions rich in aspartic acid, are activated in an ABA-dependent manner [11,12,13]. Such ABA-dependent SnRK2 activation is mediated by the soluble ABA receptors PYRABACTIN RESISTANCE (PYR/PYL)/REGULATORY COMPONENT OF ABA RECEPTOR (RCAR) and its co-receptor clade A of type 2C protein phosphatases (PP2C) [16,17,18,19] (Figure 2). PYR/PYL/RCARs (hereafter referred as PYLs) are a family of proteins of about 150–200 amino acids in length that share a conserved Steroidogenic Acute Regulatory protein (StAR)-related lipid transfer (START) domain [16]. The Arabidopsis genome contains 14 members of PYL proteins: PYR1 and PYR1-like 1 (PYL1)-PYL13 [16] or RCAR1-RCAR14 [17]. PP2Cs are monomeric serine/threonine protein phosphatases and of the 76 genes predicted to encode PP2Cs in Arabidopsis, 9 members are categorized into clade A [72]. Based on sequence similarity, clade A PP2Cs can be classified into two subfamilies, a group consisting of ABI1, ABI2, HYPERSENSITIVE TO ABA 1 (HAB1) and HAB2 and a group containing AHG1, AHG3/PROTEIN PHOSPHATASE 2CA (PP2CA), HIGHLY ABA-INDUCED PP2C GENE 1 (HAI1), HAI2 and HAI3 [73]. At least six of clade A PP2Cs (ABI1, ABI2, HAB1, HAB2, AHG1 and AHG3) function as negative regulators of ABA-dependent SnRK2 activation [18,19,74]. ABI1-type PP2Cs localize to both the cytosolic region and nucleus and are involved in the broad ABA responses [18,75]. In contrast, AHG1 and AHG3 mainly localize to the nucleus [18] and specifically affect ABA-related seed germination phenotypes [73,76,77,78]. In the absence of ABA, clade A PP2Cs interact with the ABA-box at the C-terminal region of subclass III SnRK2s and inactivate SnRK2s by dephosphorylating serine residues within the activation loop (corresponding to Ser-175 in AtSRK2E), thereby keeping the ABA signaling inactive [18,19]. In the presence of ABA, ABA binds to the hydrophobic binding pocket of the START domain of PYLs, triggering a conformational change in the PYL that reveals an interaction surface for PP2Cs [79]. Then, the ABA-PYL complex competitively interacts with PP2Cs and release SnRK2s [16,17,18,19]. The released SnRK2s then phosphorylate various downstream substrates, such as AREB/ABFs, SLAC1 and SNS1, to induce ABA- or stress-responsive gene expression, stomatal closure or the other ABA responses [14,45,46,47,48,51] (Figure 2).

The ABA signaling “core components” PYL, PP2C and SnRK2 also associate with additional regulatory proteins. For example, ARABIDOPSIS EL1-LIKE (AEL) casein kinases phosphorylate PYLs to promote their ubiquitination and degradation, resulting in suppressed ABA responses [80]. CYTOSOLIC ABA RECEPTOR KINASE 1 (CARK1), a putative receptor-like cytoplasmic kinase, phosphorylates PYR1 and PYL8 to positively regulate ABA signaling [81]. Under optimal growth conditions, TARGET OF RAPAMYCIN (TOR) kinase-mediated phosphorylation of PYLs disrupts PYL association with ABA and with PP2Cs, leading to inactivation of SnRK2s [25]. Arabidopsis E3 ubiquitin ligases PLANT U-BOX 22 (PUB22) and PUB23 promote PYL9 degradation to negatively regulate drought tolerance [82]. Arabidopsis ALG-2 INTERACTING PROTEIN-X (ALIX) attenuates ABA-induced stomatal closure by directly binding to PYLs in late endosomes, promoting their degradation [83]. In addition to PYLs, it has been reported that PP2Cs associate with several proteins. For example, Arabidopsis ENHANCER OF ABA CO-RECEPTOR 1 (EAR1) enhances the activity of six clade A PP2Cs (ABI1, ABI2, HAB1, HAB2, AHG1 and AHG3) by interacting with and releasing the N-terminal auto-inhibition of PP2Cs, resulting in suppressed SnRK2 activity [84]. DEAD-box RNA HELICASE 8 (RH8) interacts with AHG3 and inhibits its phosphatase activity [85]. DELAY OF GERMINATION 1 (DOG1) interacts with and inhibits AHG1 and AHG3 activity to regulate seed dormancy and germination [73,78]. PYL-bound ABI1 is targeted for degradation by E3 ubiquitin ligases PUB12 and PUB13 to enhance ABA-responsive SnRK2 activation [86].

In addition, to indirect regulation of SnRK2 activity by controlling PYL-PP2C module, SnRK2s are also directly regulated by several mechanisms. GLYCOGEN-SYNTHASE KINASE-3 (GSK3)-like kinase BRASSINOSTEROID INSENSITIVE 2 (BIN2), a central negative regulator in brassinosteroid signaling, activates SRK2D and SRK2I through phosphorylation on Thr-181 in SRK2D and Thr-180 in SRK2I to enhance ABA signaling [87]. Leucine-rich repeat receptor-like kinase (LRR-RLK) BRI1-ASSOCIATED RECEPTOR KINASE 1 (BAK1) phosphorylates SRK2E to induce ABA-induced stomatal closure in guard cells [88]. SRK2E and another LRR-RLK RECEPTOR-LIKE PROTEIN KINASE 1 (RPK1), which previously reported as a positive regulator in ABA signaling, phosphorylate each other to promote ABA-mediated stomatal closure [89]. *Zea mays* CASEIN KINASE 2 (CK2) phosphorylates the ABA-box of ZmOST1, increasing its binding to PP2C and triggering ZmOST1 degradation [90]. E3 ubiquitin ligases PHLOEM PROTEIN 2-B11 (PP2-B11) [91] and HIGH EXPRESSION OF OSMOTICALLY RESPONSIVE GENES 15 (HOS15) [92] attenuate ABA signaling by promoting SRK2I and SRK2E degradation, respectively. SNRK2-INTERACTING CALCIUM SENSOR (SCS), a calcium-binding EF-hand family protein, negatively regulates SnRK2 activity [93,94]. A plasma membrane-localized PP2C phosphatase CLADE-E GROWTH-REGULATING 2 (EGR2) negatively regulate plant freezing tolerance by inhibiting SRK2E kinase activity [95]. SRK2E can also be inactivated by S-nitrosylation [96] but activated by persulfidation [97] in guard cells. By strictly controlling the ABA signaling core as described above, plants can respond flexibly to environmental changes.

Another important issue is how plants induce ABA biosynthesis after osmotic stress perception (see review by Takahashi et al. [98]) and recent studies suggested that several protein kinases could also be involved in this process. Notably, under osmotic stress conditions, plants first recognize limiting water conditions in roots and transmit the stress signal(s) from roots to shoots. Indeed, ABA accumulation occurs predominantly in the vasculature of the leaves because almost all ABA synthesis-associated enzymes are expressed in vascular tissues [99]. The key enzyme for osmotic stress-induced ABA biosynthesis is NINE-CIS-EPOXYCAROTENOID DIOXYGENASE 3 (NCED3), whose transcripts are highly increased in response to osmotic stress perception [100,101]. The resulting increased levels of NCED3 promotes ABA biosynthesis and the accumulated ABA subsequently spreads from the vascular tissues to all tissues to promote gene expression and stomatal closure as a means to promote osmotic stress tolerance. A recent study reported that dehydration stress induces the activation of NGATHA1 (NGA1), a transcription factor that directly binds to the *NCED3* promoter to enhance its expression [102]. Such osmotic stress-induced NGA1 activation probably require phosphorylation of NGA1 because mutations of *NGA1* that result in phospho-mimetic substitutions of NGA1 caused altered transcriptional activity [102]. However, no protein kinase responsible for phosphorylating NGA1 has been identified to date. Another study reported that CLAVATA3/EMBRYO-SURROUNDING REGION RELATED 25 (CLE25) peptide transmits water-deficiency signals from roots to shoots for ABA biosynthesis in leaves [103]. Root-derived CLE25 peptides are perceived by plasma membrane-localized receptor-like kinases BARELY ANY MERISTEM 1 (BAM1) and BAM3 and enhance ABA biosynthesis through induction of the *NCED3* expression to induce stomatal closure [103]. In addition, another signal peptide CLE9 is required for the ABA biosynthesis and stimulates stomatal closure through OST1- and MPK3/MPK6-mediated pathway [104]. Further analyses will be required for understanding the physiological and molecular relationship among such signaling components in ABA biosynthesis in response to osmotic stress.

### 3.2. Group B Raf-Like Protein Kinases

All SnRK2 protein kinases in Arabidopsis, except for SRK2J, are activated in response to osmotic stress [12] and this response is collectively referred to as osmotic stress-induced or ABA-independent SnRK2 activation. In addition to Arabidopsis, ABA-independent SnRK2 activation was reported in several plant species including *Oryza sativa* [13], *Nicotiana tabacum* [105], *Glycine max* [106] and *Physcomitrella patens* [54]. Although ABA accumulates after osmotic stress perception and the PYL-ABA-PP2C complexes mediate the ABA-dependent SnRK2 activation [16,17,18,19], osmotic stress-induced SnRK2 activation were still observed in the ABA-deficient and ABA-insensitive mutants [11,40], indicating that SnRK2 activation occurs independently of ABA accumulation under these conditions. SnRK2 may be activated via auto-phosphorylation on a conserved serine residue within its activation loop (Ser-175 for SRK2E), which is essential for SnRK2 catalytic activity [39,74] and is also a known target residue of PP2C activity [18,19,107]. However, accumulating evidence suggests that additional mechanisms may be involved in SnRK2 activation. First, crystal structure analyses revealed that the activation loop of SRK2D and SRK2I are less stable than that of SRK2E, indicating that SRK2D/I may need to be phosphorylated by other unknown upstream kinases for full activation [108]. Second, staurosporine, a general kinase inhibitor, could not block osmotic stress-induced SnRK2 activation in vivo even though staurosporine blocks SnRK2 activity in vitro [40], suggesting that staurosporine-insensitive protein kinase(s) may be involved in SnRK2 activation. Moreover, no clear SnRK2 hyper-activation was observed in higher-order *pp2c* mutants under optimal growth conditions [69,95], indicating that additional regulatory steps are needed for SnRK2 activation during osmotic stress responses. Importantly, in addition to the phosphorylation on Ser-175 in SRK2E, Vlad et al. found that an additional phosphorylation site, corresponding to Ser-171 in the activation loop of SRK2E, is required for SnRK2 activation *in planta* [74].

A breakthrough in understanding of SnRK2 activation was made in the moss *P. patens.* In this research, ABA-insensitive mutants were isolated from a UV-irradiated mutant population and one of these mutants named AR7 showed drastically reduced ABA sensitivity, osmotic and freezing tolerance [20]. The mutation in the AR7 mutant was mapped to a gene encoding a subgroup B3 Raf-like protein kinase (B3-Raf) named “*ABA AND ABIOTIC STRESS-RESPONSIVE RAF-LIKE KINASE* (*ARK*)” [20], also known as “*ABA NON-RESPONSIVE* (*ANR*)” [109] or “*CONSTITUTIVE TRIPLE RESPONSE 1-LIKE* (*CTR1L*)” [110]. SnRK2 activation was drastically impaired in AR7, when exposed to ABA or multiple abiotic stress (i.e., cold, desiccation and osmotic stress) [20], suggesting ARK acts as an upstream kinase of SnRK2. Consistent with this hypothesis, ARK directly phosphorylated PpSnRK2B in vitro on two conserved serine residues (Ser-165 and Ser-169), corresponding to Ser-171 and Ser-175 in AtSRK2E [20]. Large-scale transcriptome and phosphoproteome analyses between WT and AR7 mutant further confirmed the importance of ARK in SnRK2 activation, with ~90% of both ABA-responsive transcripts [20] and ABA-responsive phosphopeptides [111] regulated in an ARK-dependent manner.

In 2020, four groups independently reported that group B Raf-like protein kinases regulate SnRK2s in Arabidopsis [21,22,23,24] and their work was reviewed recently [70,112,113,114]. Of the 48 Raf-like protein kinases encoded in the Arabidopsis genome, 22 genes are categorized as group B and further classified into four subgroups: B1 (three genes), B2 (six genes), B3 (six genes) and B4 (seven genes) [115]. The study by Katsuta et al. [21] focused on three Arabidopsis B3 kinases (Raf4/AtARK1, Raf5/AtARK2 and Raf6/AtARK3), all of which can complement the moss ARK function in the cross-species complementation [20]. The T-DNA insertional triple knockout mutant (*Atark* TKO) showed reduced ABA-sensitivity at the cotyledon greening stage and impaired responses to osmotic stress, including stress-responsive gene expression and stomatal closure. Among SRK2E, SRK2C and SRK2G, AtARK1 prefers subclass III SRK2E as a substrate and directly phosphorylated the two serine residues (Ser-171 and Ser-175) in SRK2E in vitro. Intriguingly, in *Atark* TKO, osmotic stress-induced activation of subclass III SnRK2s was clearly reduced, whereas ABA-induced SnRK2 activation was still observed, indicating that AtARKs are required specifically for osmotic stress-induced SnRK2 activation. In addition, 60–70% of osmotic stress- and AREB/ABF-regulated genes [46] overlapped with osmotic stress- and AtARK-regulated genes [21], demonstrating that AtARKs mediate the subclass III SnRK2–AREB/ABF pathway (Figure 2). Takahashi et al. [22] also focused on B3-type Raf kinases from ABA-insensitive mutant screenings using artificial miRNA (amiRNA) expressing transgenic lines. In addition to the *amiRNA* lines, both T-DNA insertions and CRISPR/Cas9 mediated-mutations were used to generate mutations in genes encoding *Raf3*/*M3Kδ1*, *Raf4*/*M3Kδ7*/*AtARK1* and *Raf5*/*M3Kδ6*/*AtARK2*. The resulting triple mutant showed reduced ABA sensitivity and osmotic stress responses and less SnRK2 activity. Notably, phosphorylation of Ser-171 in SRK2E by B3-Raf kinases is necessary for autophosphorylation-mediated SRK2E re-activation after dephosphorylation by PP2Cs [22]. SRK2E-mediated anion channel SLAC1 activation also required B3-Raf kinases, suggesting that B3-Raf kinases are essential components in some subclass III SnRK2s-mediated ABA responses (Figure 2). Soma et al. [23] focused on the upstream activation mechanisms of subclass I-type SnRK2s, which rapidly activate in response to osmotic stress independently of ABA. Using liquid chromatography–tandem mass spectrometry (LC-MS/MS), three B4-Raf kinases (Raf18, Raf20 and Raf24), all of which were previously reported to be phosphorylated during early osmotic stress responses [116], were identified as SRK2A- and/or SRK2G-interacting proteins [23]. These interactions occurred within processing-bodies (P-bodies) during osmotic stress. Under drought conditions, a T-DNA insertional triple knockout mutant (*raf18*/*20*/*24*) displayed the same growth retardation phenotype as a quadruple *srk2abgh* mutant that lacks all subclass I SnRK2s. Additionally, Raf20 directly phosphorylated the specific serine residue within the activation loop of SRK2G (Ser-154) and the osmostress-induced subclass I SnRK2 activation was significantly reduced in *raf18*/*20*/*24*, suggesting the essential roles of B4 Raf-like protein kinases in the activation of the subclass I SnRK2–VARICOSE pathway [61] (Figure 2). Lin et al. [24] discovered that two groups of protein kinases with molecular weights of about 100 and 130 kDa are rapidly activated in response to osmotic stress and named as OSMOTIC STRESS-ACTIVATED PROTEIN KINASES OK^100^ and OK^130^. Using a phosphoproteomic approach and CRISPR/Cas9 to generate high-order *raf* mutants, the authors revealed that B2/B3-Raf kinases correspond to OK^100^ and are responsible for the activation of subclass III SnRK2s, while B4-Raf kinases correspond to OK^130^ and are responsible for the activation of subclass I SnRK2s under osmotic-stress conditions (Figure 2). Importantly, the higher-order *raf* mutants among B2, B3 and B4 Rafs displayed strong growth defects even under normal conditions, suggesting that Raf-like protein kinases are required for proper plant growth and development as well as osmotic stress tolerance. Intriguingly, further recent study by Lin et al. [117] supported the importance of B2/B3 Raf-like kinases in ABA signaling. High-order mutants among B2/B3-Rafs (*OK^100^-oct* or *OK^100^-nonu* lacking 8 and 9 members, respectively) are less sensitive to ABA at seed germination and cotyledon greening and in ABA-responsive gene expression [117]. In in vitro kinase assays with SRK2E as a substrate, B2-Rafs preferred to phosphorylate Ser-171, whereas B3-Rafs preferred to phosphorylate Ser-175 [117]. Consistent with Takahashi et al. [22], B2/B3 Raf-mediated trans-phosphorylation of SnRK2s was crucial for SnRK2 auto-activation, suggesting that phosphorylation of SnRK2 by B2/B3-Rafs can initiate activation of subclass III SnRK2s [117]. Taken together, recent investigations revealed that the group B3 Raf-like protein kinase - subclass III SnRK2 module is evolutionarily conserved across 400 million years of land plants evolution for conferring osmotic stress tolerance [21,22,24,117]. In addition, higher plants have subsequently developed a novel B4 Raf-like protein kinase—subclass I SnRK2 system, perhaps to cope with osmotic stress conditions independently of ABA-mediated PYL-PP2C regulation [23,24,54] (Figure 2).

## 4. SnRK2—TOR Signaling Module Balances Growth and Stress Responses

In addition to the induction of stress tolerance under unfavorable conditions, it is important to understand how plants maintain a balance between growth and stress responses as well. Under abiotic stress conditions such as drought, plants spontaneously suppress growth to cope with limited energy-availability conditions caused by reduced photosynthetic rate [71]. That is, plants transiently sacrifice growth to facilitate protective stress responses. Recent investigations proposed that both the core components of ABA signaling and TARGET OF RAPAMYCIN (TOR)-mediated signaling play important roles in a balance between growth and stress responses (see excellent review by Fu et al. [27]). TOR is an atypical serine/threonine protein kinase that is evolutionarily conserved in almost all eukaryotes, including animals and plants and is a positive regulator of growth and development [118,119]. TOR exerts its function in complex with other proteins and Arabidopsis TOR complex is composed of TOR, REGULATORY-ASSOCIATED PROTEIN OF TOR (RAPTOR) and LETHAL WITH SEC THIRTEEN 8 (LST8) [27] (Figure 2). RAPTOR may act as a scaffold to facilitate TOR-binding to other proteins such as RIBOSOMAL-PROTEIN S6 KINASES (S6K) that are crucial regulators of protein translation [120]. On the other hand, LST8 interacts with the C-terminal kinase domain of TOR and may inhibit TOR kinase activity towards selective substrates [121]. AtTOR is encoded by a single-copy gene, whose expression pattern is limited in endosperm, embryo and primary meristems and the null *tor* mutant is embryo lethal [122]. Two-copies of *RAPTOR* (*RAPTOR1A* and *RAPTOR1B*) exists in the Arabidopsis genome and the double mutant of *AtRAPTORs* (*raptor1a/b*) shows normal embryonic development but is unable to maintain post-embryonic seedling growth [123]. Arabidopsis contains two copies of *LST8* (*LST8-1* and *LST8-2*), but only *LST8-1* is significantly expressed [121]. The *lst8-1* mutant displays modest dwarf and early senescence phenotypes [121]. Recent studies have shed light on the crosstalk between TOR complex-mediated signaling and phytohormone signaling during plant growth promotion. For example, sugar-activated TOR can promote hypocotyl elongation in darkness through autophagic regulation of the transcription factor BRASSINAZOLE-RESISTANT 1 (BZR1), a positive regulator of the brassinosteroid signaling [124] (Figure 2). In addition, TOR directly phosphorylates and stabilizes PIN-FORMED 2 (PIN2), an auxin efflux carrier, thereby influencing the gradient distribution of PIN2 to promote cell expansion in roots [125]. Moreover, ETHYLENE INSENSITIVE 2 (EIN2), a central integrator in ethylene signaling, is a direct substrate of TOR [126] (Figure 2). TOR-mediated EIN2 phosphorylation prevents its nuclear shuttling, thereby inhibiting EIN2-mediated negative regulation of hypocotyl cell elongation [126] (Figure 2).

Recently, a reciprocal negative feedback loop between TOR signaling and ABA signaling has been shown to regulate a balance between growth and protective stress responses in Arabidopsis. Under optimal growth conditions, TOR phosphorylates ABA receptor PYLs at a conserved serine residue in PYL family (corresponding to Ser-114 in PYL4 and Ser-119 in PYL1) [25]. This phosphorylation site is located within the ABA binding pocket [79]. Therefore, TOR-mediated phosphorylation of PYLs activates PP2C phosphatases by inhibiting PYL-binding to ABA, thereby compromising SnRK2-mediated ABA signaling [25] (Figure 2). Consistent with this notion, mutants lacking functional components of the Arabidopsis TOR complex, such as *raptor1b*, *lst8-1* and estradiol-inducible *TOR* RNAi line (*es-tor*), are more sensitive to exogenous ABA treatment compared to wild-type plants [25,127]. On the other hand, ABA signaling also represses TOR signaling. Under unfavorable conditions, osmotic stress or increased ABA trigger SnRK2 activation. In turn, activated SnRK2s directly interact with and phosphorylate RAPTOR1B to dissociate it from TOR complex, thereby inhibiting TOR kinase activity [25]. Therefore, under optimal growth conditions, active TOR promotes growth with repressing ABA signaling, while under drought conditions, activated SnRK2s promote protective stress responses with repressing TOR-mediated growth promotion (Figure 2). Notably, in this model, SnRK2 is supposed to act antagonistically with growth during stress response, whereas another recent study revealed that SnRK2s also have the aspect of promoting growth under nutrient-rich conditions by facilitating TOR signaling through SnRK1 regulation [26]. In that model, SnRK2 and SnRK1 form a complex via interaction with PP2C under optimal growth conditions, thereby inhibiting the interaction between SnRK1 and TOR complex and maintaining TOR kinase activity to promote growth [26]. Under stressed conditions, SnRK1 is released from the sequestration by SnRK2-PP2C complex and inhibits TOR-mediated signaling to repress growth [26] (Figure 2). Collectively, these studies demonstrated that both TOR signaling and ABA/SnRK2 signaling are important for proper growth regulation during osmotic stress responses.

In addition to the regulation of TOR complex, recent studies have reported that SnRK2 can modulate some cellular processes under non-stressed conditions. For example, subclass III SnRK2s modulate metabolism and leaf growth under optimal growth conditions through regulation of the tricarboxylic acid (TCA) cycle [128]. In addition, although no detectable CO_2_- or methyl jasmonate (MeJA)-induced activation of SRK2E/OST1 in guard cells was observed, a basal level of OST1/SRK2E activity is required for the both CO_2_- and MeJA-induced stomatal closure [129,130]. Intriguingly, recent study successfully detected such a basal OST1/SRK2E kinase activity using Förster resonance energy transfer (FRET)-based biosensor [131]. Further analyses will be required to understand how basal SnRK2 activity regulates several cellular processes under non-stressed conditions.

## 5. Conclusions and Future Perspectives

Under osmotic stress conditions, SnRK2-type protein kinases are activated in the both ABA-dependent and -independent manner and play essential roles in the induction of multiple osmotic stress responses. In addition to the mechanisms of PYL-PP2C complex-mediated ABA-dependent activation, additional mechanisms of osmotic stress-induced or ABA-independent SnRK2 activation has been unveiled recently. Following the identification and characterization of ARK in *P. patens*, Arabidopsis group B Raf-like protein kinases (group B Raf) were identified as essential components that integrate early osmotic stress signaling into osmotic stress-induced SnRK2 activation. The next challenge is to identify upstream components that activate group B Raf during early osmotic stress responses. Notably, *P. patens* ARK/CTR1L [110] and Arabidopsis B3 kinase CONSTITUTIVE TRIPLE RESPONSE 1 (CTR1) [132] physically interact with ethylene receptors. On the other hand, *P. patens* ARK is rapidly activated after ABA treatment [133], while no clear ABA-induced activation of Arabidopsis group B Raf were observed [24,117]. This difference suggests that the regulatory mechanisms upstream of Raf-like kinases may have been altered during evolution. In addition, the crosstalk between ABA-dependent (PYL-PP2C) and ABA-independent (group B Raf) SnRK2 activation pathway will be an important area of future research. In this regard, a recent study in Arabidopsis showed that ABA-dependent SnRK2 activation was reduced, but ABA-independent SnRK2 activation was enhanced, in a higher-order *PYLs* mutant, indicating that PYL-mediated ABA signaling antagonizes ABA-independent SnRK2 activity [134]. Additionally, another recent study reported that co-treatment of osmotic stress and exogenous ABA led to chlorosis in Arabidopsis [135]. Such a complicated crosstalk may indicate that a balanced ABA signaling is important for proper growth under osmotic stress conditions. In addition to group B Raf, several other upstream kinases, such as BIN2 [87], BAK1 [88] and RPK1 [89], may also be involved in the activation of SnRK2s. Understanding the interplay between these protein kinases and group B Raf in osmotic stress-induced SnRK2 activation will need further studies.

In addition to the roles of SnRK2 during osmotic stress responses, recent studies also have highlighted the roles of SnRK2 in the plant growth regulation under optimal conditions [25,26]. These studies demonstrated that TOR complex-mediated growth promotion and ABA/SnRK2 module-mediated protective stress responses function antagonistically. However, it is noteworthy that endogenous ABA is crucial for proper growth because ABA biosynthesis mutants display reduced growth even under normal conditions [136]. In addition, plants defective in AtTOR complex components, such as the mutants *raptor1b* and *lst8-1*, as well as wild-type seedlings treated with the TOR inhibitor AZD-8055 [127], accumulate decreased levels of ABA under normal conditions, suggesting the involvement of TOR signaling in ABA biosynthesis. How basal level of ABA signaling and TOR-signaling contribute plant growth is an important question for future studies.

## Data Availability

The data is contained within the article.
